# The Role of Mast Cells in Parathyroid Bone Disease

**DOI:** 10.1002/jbmr.49

**Published:** 2010-01-29

**Authors:** Russell T Turner, Urszula T Iwaniec, Kevin Marley, Jean D Sibonga

**Affiliations:** 1 Department of Nutrition and Exercise Sciences, Oregon State UniversityCorvallis, OR, USA; 2 Division of Space Life Sciences, Universities Space Research AssociationHouston, TX, USA

**Keywords:** hyperparathyroidism, iliac crest bone biopsy, PDGF, PI3K, stem cell factor

## Abstract

Chronic hyperparathyroidism (HPT) is a common cause of metabolic bone disease. These studies investigated the underlying cellular and molecular mechanisms responsible for the detrimental actions of elevated parathyroid hormone (PTH) on the skeleton. Bone biopsies from hyperparathyroid patients revealed an association between parathyroid bone disease and increased numbers of bone marrow mast cells. We therefore evaluated the role of mast cells in the etiology of parathyroid bone disease in a rat model for chronic HPT. In rats, mature mast cells were preferentially located at sites undergoing bone turnover, and the number of mast cells at the bone–bone marrow interface was greatly increased following treatment with PTH. Time-course studies and studies employing parathyroid hormone–related peptide (PTHrP), as well as inhibitors of platelet-derived growth factor-A (PDGF-A, trapidil), *kit* (gleevec), and PI3K (wortmannin) signaling revealed that mature mast cell redistribution from bone marrow to bone surfaces precedes and is associated with osteitis fibrosa, a hallmark of parathyroid bone disease. Importantly, mature mast cells were not observed in the bone marrow of mice. Mice, in turn, were resistant to the development of PTH-induced bone marrow fibrosis. These findings suggest that the mast cell may be a novel target for treatment of metabolic bone disease. © 2010 American Society for Bone and Mineral Research.

## Introduction

Hyperparathyroidism (HPT), a common endocrine disorder, results in a spectrum of skeletal abnormalities ranging from increased bone turnover in individuals with mild (asymptomatic) HPT to parathyroid bone disease in patients with severe chronic HPT.(1,2) In individuals with severe primary or secondary chronic HPT, bone marrow–derived preosteoblasts exhibiting a fibroblast-like morphology (fibroblasts) migrate to bone surfaces, where they produce copious quantities of poorly organized extracellular matrix, which is a hallmark of high-turnover parathyroid bone disease. Defects in the organization and mineralization of bone matrix and focal bone resorption contribute to poor bone structural quality and thus result in increased fracture risk.(1–3) Moreover, HPT patients with severe focal bone resorption often experience debilitating bone pain.(4)

In addition to the high-turnover parathyroid bone disease just noted, mixed and low-turnover forms of the disease have been described. However, low-turnover disease may be due to treatment modalities and not the disease per se.(5) Although focal bone resorption, osteomalacia, and peritrabecular bone marrow fibrosis (osteitis fibrosa) have been reported in bone biopsies from patients with severe HPT,(6) our understanding of the etiology of the disease has been hampered by the relatively small number of individuals who have been studied, variable duration and severity of the disease, and the confounding effects of treatment modalities. As a result, the natural history of disease progression and the relationship among the abnormal bone resorption, matrix synthesis, and mineralization are incompletely understood.

Chronically elevated secretion of PTH typically occurs as a consequence of a dysfunction in the parathyroid gland (primary HPT) or, alternatively, in response to chronic hypocalcemia and/or hyperphosphatemia (secondary HPT). Severe parathyroid bone disease, in developed countries, is most often associated with abnormities in calcium and phosphorous metabolism caused by renal failure. The respective role of elevated PTH, increases or decreases in serum calcium or phosphorous, or uremia to the presentation of parathyroid bone disease is not well defined. Partial (5/6) nephrectomy induces mild to moderate (stage 3) chronic renal insufficiency in animals.(7) The slow and variable rate of skeletal disease progression is a major disadvantage of this model. We therefore developed a model for severe HPT in which PTH is continuously administered using a subcutaneous osmotic pump.(8) Within 1 week, the rats developed a skeletal pathology characterized by elevated bone turnover, defective matrix mineralization, and severe peritrabecular fibrosis. Importantly, the skeletal abnormalities observed following treatment with continuous PTH (cPTH) were virtually identical to those in patients undergoing renal dialysis reported here and in smaller studies.(9,10) Although the mechanisms underlying primary and secondary HPT differ, the skeletal consequences are similar (ie, osteitis fibrosa, elevated bone turnover, and defective bone matrix mineralization). These similar skeletal responses suggest that an elevated PTH level is the most important factor in the etiology of high-turnover parathyroid bone disease.

Animal studies have revealed that chronically elevated serum PTH increases proliferation and enhances migration of early preosteoblastic fibroblasts to bone surfaces, induces bone lining cells to synthesize bone matrix, and has variable effects on osteoblast apoptosis.(11–15) Preosteoblastic fibroblasts recruited to bone surfaces normally differentiate to osteoblasts, but terminal differentiation is impaired by the presence of continuously elevated PTH.(14)

Whole-genome studies identified overexpression of platelet-derived growth factor-A (PDGF-A) as a candidate causal factor for fibroblast migration to bone surfaces in a rat model for chronic HPT.(16) PDGF-A has been shown to play a role in the etiology of fibrosis during tissue regeneration and a wide variety of pathologic conditions, including bone marrow fibrosis in patients with myeloproliferative diseases.(17–22) The hypothesis that increased PDGF-A levels in response to HPT contribute to the disease is strengthened by the finding that trapidil, a PDGF-A signaling antagonist, inhibits the development of osteitis fibrosa in the rat model for chronic HTP.(16) Trapidil was shown to blunt PTH-induced increases in the expression of several profibrotic genes, including *lysyl oxidase*.(23) To our surprise, immunostaining identified peritrabecular mast cells in PTH-treated rats as intensely positive for PDGF-A and lysyl oxidase.(16,23) Thus mast cell–derived proinflammatory cytokine or enzyme secretion may mediate peritrabecular fibrosis by activating preosteoblastic fibroblast proliferation and migration. However, a mechanism by which mast cells, which are normally located throughout the marrow cavity, could lead to the peritrabecular localization of bone marrow–derived fibroblasts is lacking. The studies described herein investigated factors important in mast cell chemotaxis and survival. The results suggest that mast cell accumulation onto bone surfaces in response to elevated PTH and their interaction with osteoblast lineage cells via pathways involving PDGF receptor-α, c-*kit*, and PI3K signaling precede and contribute to PTH-induced peritrabecular fibrosis.

## Methods

### Studies in humans

#### Clinical chart review

The chart review was approved by the Institutional Review Board at the Mayo Clinic and conducted in accordance with Declaration of Helsinki principles. The purpose of the chart review was to determine the frequency of osteitis fibrosa and the association of this condition with other skeletal abnormalities in iliac crest biopsies of patients diagnosed as having HPT (*n* = 605, bone biopsies taken at the Mayo Clinic, Rochester, MN, USA, from 1983 to 2000; Table 1 shows the age distribution). The biopsies were evaluated as described previously.(24) A Mayo hematopathologist diagnosed the presence of fibrosis in this cohort of HPT patients. Fibrosis, indicative of osteitis fibrosa, is not present in iliac crest bone biopsies of healthy individuals. The diagnosis of additional skeletal abnormalities in HPT patients was based on quantitative histomorphometry in which the patients values were compared with values obtained from healthy female volunteers (*n* = 18), a reference database used by the Mayo Bone Histomorphometry Laboratory from 1983 to 2004. This database was updated in 2004 to include 43 men and to increase the number of women to 46. There were no sex-specific differences in histomorphometric endpoints in the updated database that would affect interpretation of the original diagnosis of skeletal abnormalities in patients diagnosed with HPT. The bone turnover measurements evaluated consisted of osteoclast number, eroded perimeter, osteoid perimeter, and bone-formation rate.

**Table 1:** Age Distribution of Patients Diagnosed as Having HPT

Age range (years)	Number
2–19	14
20–29	38
30–39	82
40–49	101
50–59	120
60–69	129
70–79	46
80–84	2
Age not recorded	73

#### Effect of HPT on mast cell number

A retrospective analysis was performed on iliac crest biopsies in a subset (30 females) of the patients diagnosed with chronic renal failure and compared with 20 healthy women of similar age.

### Studies in rats

Similar to humans, parathyroid bone disease is induced by chronic elevations in PTH levels in male and female rats over a wide range of ages. Sprague-Dawley rats ranging in age from 6 weeks to 6 months, depending on experiment, were used in the rat studies. The animals were obtained from Harlan Sprague-Dawley, Inc. (Indianapolis, IN, or Madison, WI, USA) and maintained under standard conditions with a 12-/12-hour light/dark cycle. Food (Laboratory Rodent Diet 5001, containing 0.95% calcium, 0.67% phosphorus, and 4.5 IU/g of vitamin D_3_; LabDiet, St. Louis, MO, USA) and water were provided *ad libitum* to all rats. The animals were maintained in accordance with the NIH *Guide for the Care and Use of Laboratory Animals*, and the experimental protocols were approved by the Institutional Animal Care and Use Committee. For administration of cPTH, rats were implanted subcutaneously (sc) with osmotic pumps (Alza Corp., Mountain View, CA, USA) delivering vehicle or 40 µg/kg per day of human PTH(1-34) (Bachem, Torrance, CA, USA). Although hypercalcemic, the rats tolerated continuous PTH well. For tissue collection, all rats were anesthetized with ketamine (50 mg/kg) and xylazine HCl (5 mg/kg), and death was induced by exsanguination followed by cardiac excision. Unless specified otherwise, bones (tibias or femurs) were removed at death and fixed in 70% alcohol for bone histomorphometry.

#### Effects of cPTH on the number and distribution of bone marrow mast cells

Six-month-old female rats were divided into two treatment groups (*n* = 8 rats/group): (1) vehicle or (2) cPTH. cPTH was administered as described earlier. Animals were euthanized after 7 days of treatment.

#### Effects of cPTH on bone ultrastructure

This study was performed to confirm the histologic identification of mast cells on bone surfaces following administration of cPTH. Three-month-old female rats were randomized into two treatment groups (*n* = 10 rats/group): (1) vehicle or (2) cPTH. Animals were euthanized after 7 days of treatment. Left tibias were processed for transmission electron microscopy (TEM).

#### Role of cell proliferation in cPTH-induced mast cell accumulation onto peritrabecular bone surfaces

Six-month-old female rats were divided into two groups (*n* = 3 rats/group): (1) vehicle + [^3^H]thymidine or (2) cPTH + [^3^H]thymidine. The rats were implanted sc with osmotic pumps containing 1.5 mCi [methyl-^3^H]thymidine (specific activity 90 Ci/mmol; Amersham Pharmacia Biotech, Piscataway, NJ, USA) in aqueous solution with 2% ethanol for 1 week to label the DNA of all cells that progress through the cell cycle.(11) The rats were coinfused with vehicle or PTH and euthanized after 7 days of treatment. Femurs were removed and fixed in 10% neutral buffered formalin overnight for radioautography, as described previously.(11)

#### Effects of continuous sc infusion of PTHrP on mast cell recruitment to bone–bone marrow interface and peritrabecular fibrosis

PTHrP binds to the same receptor as PTH, but elevated PTHrP is rarely associated with bone marrow fibrosis. We therefore evaluated whether continuous infusion of PTHrP results in peritrabecular localization of mast cells in rats. Six-week-old male rats were divided into three groups (*n* = 6 rats/group): (1) vehicle, (2) PTHrP at 20 µg/kg per day, or (3) PTHrP at 80 µg/kg per day. PTHrP was infused using sc implanted osmotic pumps. The animals were euthanized after 12 days of treatment.

#### Time-course effects of cPTH on mast cell recruitment to bone surfaces

A time-course study was performed to determine the kinetics of peritrabecular localization of mast cells and fibroblasts to cancellous bone surfaces in response to cPTH. These studies were performed to determine whether mast cell recruitment to bone surfaces precedes peritrabecular fibrosis. Six-month-old female rats were divided into five treatment groups (*n* = 7 or 8 rats/group). Rats receiving cPTH were euthanized on days 1, 3, 5, and 7, whereas rats receiving vehicle were euthanized on day 7.

#### Effects of the PDGF receptor antagonist trapidil on mast cell distribution in normal rats and cPTH-induced mast cell recruitment to bone–bone marrow interface

Trapidil reduced peritrabecular fibrosis in a rat model for chronic HPT but did not decrease PTH-induced bone formation.(16) We therefore performed studies to determine the effects of trapidil on localization of mast cells to bone surfaces in normal rats and on cPTH-induced mast cell recruitment to bone surfaces. Three-month-old female rats were divided into four treatment groups (*n* = 8 rats/group): (1) control, (2) trapidil, (3) cPTH, or (4) cPTH + trapidil. The rats received daily sc injections of vehicle or 40 mg/kg per day of trapidil (Rodleben Pharma GmbH, Rodleben, Germany). The animals were euthanized on day 8. Trapidil alone or in combination with PTH had no notable detrimental side effects on the overall health of the rats.

#### Effects of the receptor tyrosine kinase inhibitor gleevec on mast cell distribution in bone marrow of normal rats and on cPTH-induced mast cell recruitment to bone–bone marrow interface

Gleevec, recently shown to inhibit peritrabecular fibrosis in a rat model for chronic HPT,(23) antagonizes signaling through the mast cell survival and chemotactic factor c-*kit*. The ligand for c-*kit* is produced by osteoblasts. We therefore investigated the effects of gleevec on localization of mast cells on bone surfaces in normal rats and on cPTH-induced translocation of mast cells to bone surfaces. The experimental design was the same as in the trapidil study, with the exception that *n* = 4 to 7 rats/group. The reduced numbers were due to gleevec toxicity, which was especially pronounced when gleevec was combined with cPTH. Gleevec was administered ip at 50 mg/kg per day.

#### Effect of the PI3K inhibitor wortmannin on cPTH-induced mast cell recruitment to bone–bone marrow interface

PTH can activate multiple cell signaling pathways. Recent studies suggest that peritrabecular marrow fibrosis in response to cPTH is mediated through PI3K and is antagonized by wortmannin.(23) We therefore performed a study to establish whether mast cell accumulation on bone surfaces in response to cPTH is antagonized by wortmannin. The experimental design was the same as for the trapidil study described earlier, with the exception that *n* = 9 rats/group. Wortmannin was administered sc at 32 mg/kg per day. Other than variable bruising at the site of administration, the rats tolerated treatment with wortmannin well.

### Studies in mice

#### Mast cell distribution in normal mice

Representative tissues from male and female growing and adult (4 to 26 weeks of age) mice were processed for evaluation of mast cells. The following mouse strains were investigated: C57BL/6, WBB6F1, and DBA.

#### Effect of cPTH on bone histomorphometry in DBA mice

The mice were divided randomly into two groups and implanted with sc osmotic pumps loaded to deliver vehicle or PTH (40 µg/kg per day) for 1 week (*n* = 5 mice/group).

### Bone histomorphometry

#### Human

Measurements were performed at a standardized site in fluorochrome-labeled unstained and Goldner-stained iliac crest bone biopsies as described previously.(24) Mast cell measurements were performed on toluidine blue–stained sections.

#### Rat

Measurements were performed at a standardized site in the proximal tibial metaphysis as described previously.(25)

#### Mouse

Measurements were performed at a standardized site in the distal femur metaphysis as described previously.(26)

For histomorphometric evaluation of cancellous bone and bone marrow fibrosis, proximal tibias (rat) or femurs (mouse) were dehydrated in graded ethanol and xylene and embedded undecalcified in modified methyl methacrylate or decalcified and embedded in either JB-4 plastic or parafin. Longitudinal sections (4 µm thick) were cut on a Reichert-Jung Supercut 2050 Microtome (Leica, Heidelberg, Germany) and affixed to slides precoated with 1% gelatin. In human studies, the sections were stained with either toluidine blue or Goldner's stain. Additional sections were left unstained and used for assessing fluorochrome-based measurements. In the rat and mouse studies, one section per animal (undemineralized sections only) was stained according to the Von Kossa method with a tetrachrome counterstain (Polysciences, Warrington, PA, USA) and used for assessing bone area and cell-based measurements. Alternatively, the JB-4 plastic– and/or paraffin-embedded sections were stained with toluidine blue. A standard sampling site with an average area of 2.7 mm^2^ (rat) or 1.6 mm^2^ (mouse) was evaluated in the secondary spongiosa of the proximal tibial metaphysis (rat) or distal femoral metaphysis (mouse). The following histomorphometric data were collected: mast cell number per bone perimeter (number of mast cells/mm) and fibroblast perimeter per bone perimeter (%).

#### Radioautography

Distal femoral metaphyses were decalcified in 5% formic acid in 10% formalin, infiltrated, and embedded in JB-4 (Polysciences, Inc.) or parafin. Then 5-µm-thick sections were cut, attached to slides, dipped into melted (40°C) 1:1 diluted Ilford K5D emulsion in water (Polysciences), air dried, and kept in a light-sealed box at 4°C for up to 2 years. Sections were developed in Kodak D-19 (Sigma, St. Louis, MO, USA) for 4 minutes, fixed in Kodak fixer (Sigma) for 4 minutes, rinsed in water for 10 minutes, and stained with toluidine blue. Cells were counted as being radiolabeled if they had numerous silver grains over the nucleus. The data were expressed as percent (labeled/total × 100).(27)

#### Electron microscopy

Biopsies 3 mm^3^ from proximal tibial metaphyses were fixed and demineralized (30 days) in 1.9% gluteraldehyde and 150 mM EDTA in 60 mM sodium cacodylate buffer, pH 7.4, at 4°C. After demineralization, sections were postfixed in osmium tetroxide. Thin sections were double stained with lead citrate and uranyl acetate according to standard methods(28) and imaged on a Philips CM12 TEM.

### Statistical analysis

For the human study, means of transformed raw biopsy data from normal volunteers were used to generate *Z*-scores for histomorphometric parameters associated with cancellous bone mass (tissue referent), turnover (perimeter referent), and fibrosis (fibroblast perimeter referent).(24) A *Z*-score in the patients diagnosed with HPT of 2 SDs or greater above or below healthy control values was considered abnormal. Differences in mast cell number between HPT patients and healthy controls were determined using a *t* test. For animal studies, differences among groups were determined using a one- or two-way ANOVA test followed by a post hoc test when appropriate (SPSS 11.5, SPSS, Inc., Chicago, IL, USA). Differences were considered significant at *p* < .05. All data are expressed as mean ± SE.

## Results

### Studies in humans

**Fig. 1:**
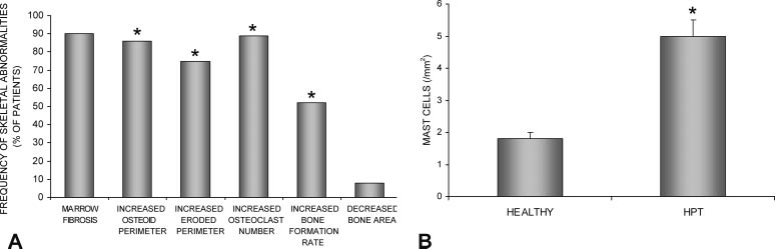
(*A*) Distribution of skeletal abnormalities in patients diagnosed with HPT (*n* = 605). Compared with healthy controls, HPT patients have marrow fibrosis and increased osteoid perimeter, eroded perimeter, osteoclast number, and bone formation rate, but most have normal cancellous bone area. Significant correlations between skeletal abnormalities and bone marrow fibrosis are indicated by an asterisk. (*B*) Mast cell number in bone biopsies from a subset of 30 women with HPT are compared with 20 healthy women of similar age. Values are mean ± SE. *p < .001.

The distribution of skeletal disorders in patients (*n* = 605; mean age 52 years, range 2 to 80 years) diagnosed as having HPT is shown in Fig. 1*A*. Ninety percent of the patients diagnosed with HPT had severe peritrabecular bone marrow fibrosis (osteitis fibrosa). Compared with healthy women, 86% of patients diagnosed with HPT had increased osteoid perimeter, 75% had increased eroded perimeter, and 89% had increased osteoclast number. Bone formation (perimeter referent) was increased in over half the patients, which may explain why only 10% had cancellous osteopenia. There were highly significant associations between osteitis fibrosa and elevated indices of bone resorption (osteoclast number and eroded perimeter) and elevated osteoid perimeter and bone formation. A significant association was not detected between osteitis fibrosa and cancellous bone mass (bone area/tissue area) in the HPT patients.

Mast cell number also was increased dramatically in iliac crest bone biopsies of women diagnosed with HPT (Fig. 1*B*). To further clarify the relationships between mast cells, PTH, and bone metabolism, additional studies were performed in rodent models.

### Studies in rats

The skeletal presentation of parathyroid bone disease in rats following continuous delivery of PTH via an osmotic pump is remarkably similar to human parathyroid bone disease and includes osteitis fibrosa, as detailed below.

#### Bone marrow mast cells in normal rats are preferentially located adjacent to bone surfaces undergoing growth or turnover

**Fig. 2:**
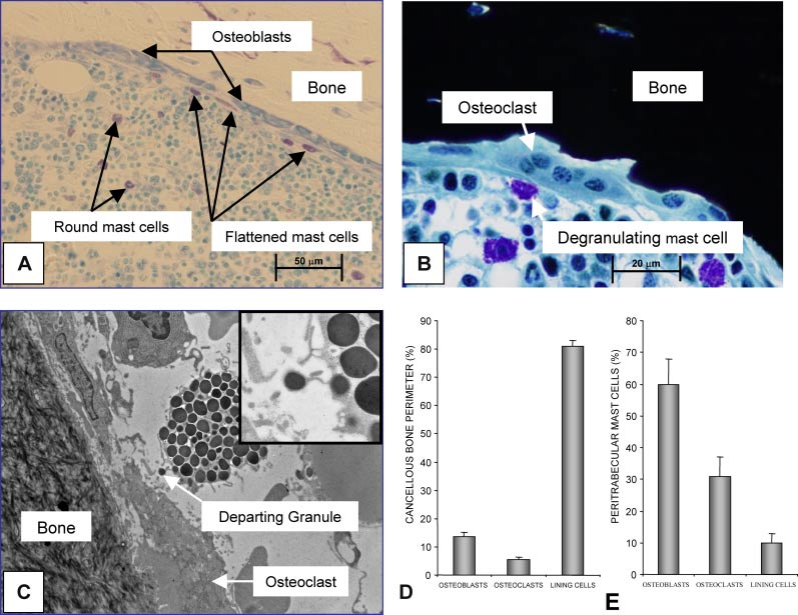
Representative bone marrow mast cells. (*A*) Light microscope image of the endocortical surface at the proximal tibia metaphysis (demineralized, embedded in JB-4 plastic, and stained with toluidine blue) in a 6-week-old normal female rat. Note the flattened appearance of the mast cells located adjacent to osteoblasts compared with the round appearance of mast cells in bone marrow. (*B*) A mast cell adjacent to an osteoclast (undemineralized bone, embedded in methyl methacrylate, and section stained according to von Kossa with tetrachrome counterstain). Note what appear to be extracellular mast cell granules. (*C*) A TEM micrograph with enlarged region from the proximal tibial metaphysis of a 3-month-old female rat treated with cPTH for 1 week shows an apparently extracellular granule from a mast cell located adjacent to an osteoclast. (*D*) Bone lining cells lined the majority of bone surface in6-month-old female rats. (*E*) In contrast, peritrabecular mast cells most often were located immediately adjacent to osteoblasts, with lesser numbers located adjacent to osteoclasts and even fewer adjacent to bone lining cells. Values are mean ± SE.

The distribution of mast cells at the bone–bone marrow interface in untreated rats is shown in Fig. 2. Mast cells located more than one cell diameter away from bone surfaces were typically round (Fig. 2*A*). In contrast, the mast cells immediately adjacent to endocortical bone surfaces lined by osteoblast lineage cells typically assumed a more flattened appearance, suggesting cell-to-cell adhesion. Mast cells lying adjacent to osteoblasts rarely exhibited evidence of substantial loss of cytoplasmic granules. In contrast, the apparent extracellular extrusion of one or a limited number of granules of mast cells located immediately adjacent to osteoclasts was common (Fig. 2*B, C*). Although highly suggestive, the extent to which such findings reflect actual degranulation occurring in vivo, as opposed to an effect of the manipulation of tissues prior to fixation, remains to be determined. The distribution of bone cells and mast cells adjacent to bone surfaces is shown in Fig. 2*D, E*, respectively. Only a small percentage of bone surface was lined by osteoblasts and osteoclasts; more than 80% of the bone surface was inactive and lined by lining cells (Fig. 2*D*). In contrast, peritrabecular mast cells were located most commonly immediately adjacent to osteoblasts, less commonly adjacent to osteoclasts, and even less commonly adjacent to bone lining cells (Fig. 2*E*), indicating a preferential distribution of mast cells at sites of bone turnover (formation or resorption).

#### PTH increases mast cell number at the bone–bone marrow interface without increasing mast cell proliferation

**Fig. 3:**
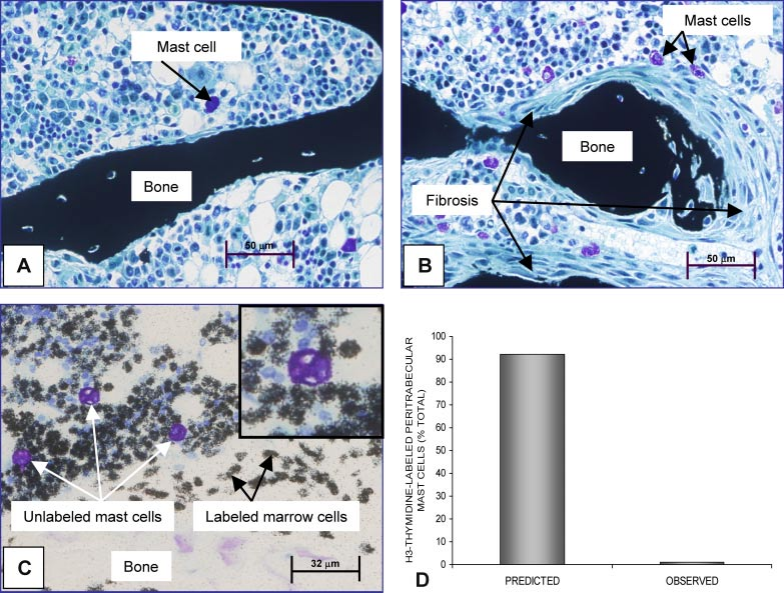
(*A*) cPTH increases mast cell number at the bone–bone marrow interface in 6-month-old female rats without increasing mast cell proliferation. Peritrabecular fibroblasts were not present, and mast cells were located predominantly in bone marrow in the control rats. (*B*) On day 7 of treatment with cPTH, most cancellous bone surfaces were covered by multiple layers of fibroblasts, and mast cells commonly were located adjacent to the fibroblasts. Specimens shown in panels *A* and *B* are undemineralized, embedded in plastic, and stained according to von Kossa with tetrachrome counterstain. (*C*) Although continuous [^3^H]thymidine labeled many bone marrow cells in 6-month-old female rats, very few mast cells were labeled (JB-4-embedded, toluidine blue–stained section). A higher-magnification insert clearly shows the absence of labeling in a representative mast cell that is surrounded by highly labeled and unlabeled marrow cells. (*D*) The predicted number of labeled mast cells assuming proliferation is required for mast cell migration to bone surfaces. The observed number was much lower, indicating that treatment with cPTH results in the migration of postproliferative mast cells to bone surfaces. The predicted value was determined as described previously.(17)

Treatment of rats for 1 week with cPTH significantly increased the number of peritrabecular mast cells (Fig. 3*A, B*). Most of the peritrabecular mast cells in cPTH-treated rats were located adjacent to fibroblasts (88%). In contrast, continuous infusion of the potent osteolytic PTH receptor ligand parathyroid hormone–related peptide (PTHrP) had no effect on mast cell distribution and did not induce osteitis fibrosa (data not shown). However, as reported previously,(29) PTHrP resulted in hypercalcemia, increased osteoclast perimeter, and cancellous osteopenia (data not shown).

We also investigated the effects of cPTH on mast cell proliferation. Cotreatment with cPTH and continuous [^3^H]thymidine labeled most bone marrow cells. However, very few (<1%) mast cells were labeled (Fig. 3*C, D*). The low number of labeled mast cells indicates that mature mast cells or precursors that can mature locally in the absence of proliferation were recruited to bone surfaces in response to cPTH.

#### Mast cell distribution and peritrabecular fibrosis

**Fig. 4:**
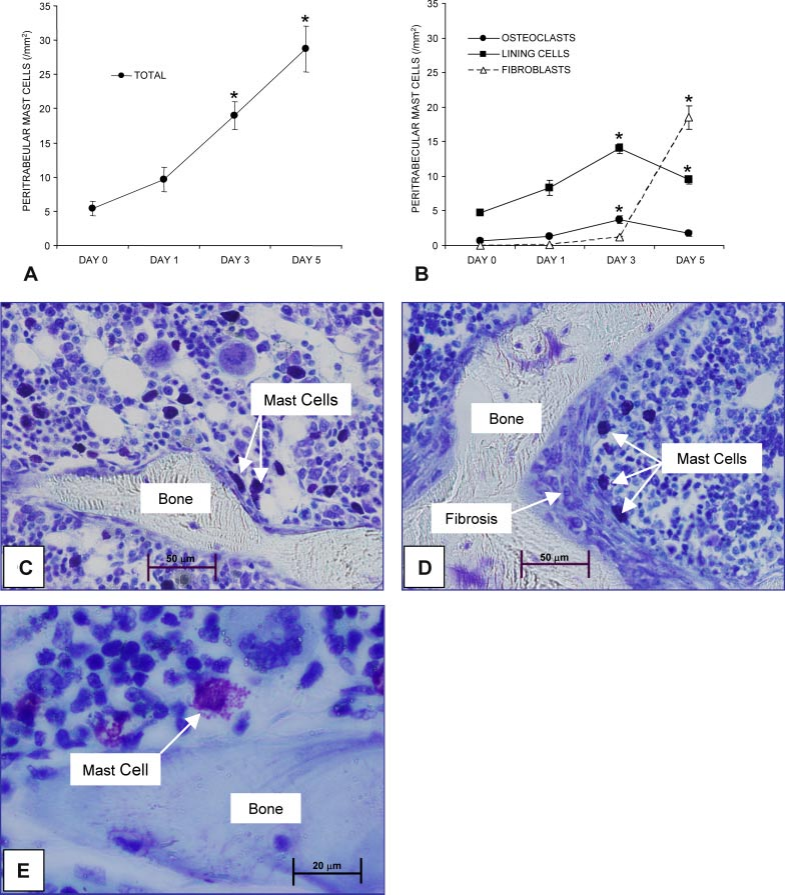
The time-course effect of cPTH on increases in and localization of marrow mast cells in relation to bone surfaces (*A*) and the association between mast cells and specific cell types on bone surfaces (*B*) in 3-month-old female rats. Note that an increase in peritrabecular mast cells was observed by day 3 (*A*, *C*) that preceded fibrosis at day 5 (*B*, *D*). A higher magnification (*E*) shows metachromatic staining of granules in a peritrabecular mast cell following treatment with cPTH for 5 days. Specimens shown in panels *C* and *D* are undemineralized and embedded in methyl methacrylate, and the specimen in panel *E* is demineralized and embedded in paraffin. All three specimens are stained with toluidine blue. Values are mean ± SE. *Different from day 0, *p* < .05.

Studies were performed to determine the time course for cPTH-induced relocalization of fibroblasts and mast cells onto cancellous bone surfaces (Fig. 4). There was a 3.5-fold increase in the number of peritrabecular mast cells 3 days following initiation of cPTH (Fig. 4*A, B*), a time point at which bone surfaces were free of fibroblasts (Fig. 4*C*). Following 5 days of treatment, there was a further increase in the number of peritrabecular mast cells. However, most of the mast cells were now located adjacent to peritrabecular fibroblasts that lined bone surfaces (Fig. 4*B, D, E*).

**Fig. 5:**
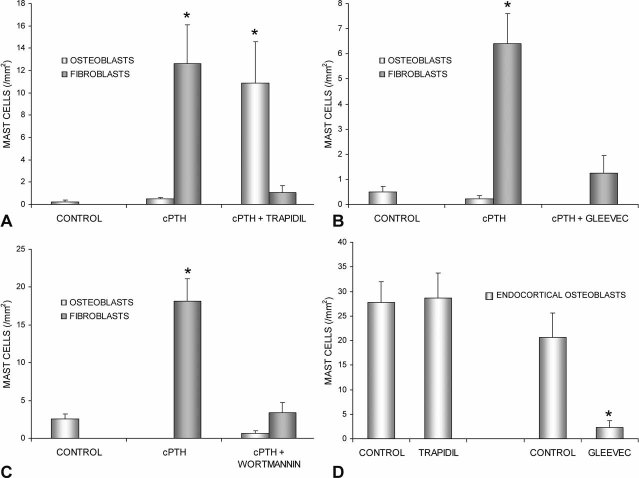
The effects of the small-molecule antagonists trapidil, gleevec, and wortmannin on mast cell number on bone surfaces of 3-month-old female rats. Continuous infusion of PTH increased the number of mast cells associated with fibroblasts (*A*). The PDGF receptor antagonist trapidil did not prevent increases in mast cells at the bone–bone marrow interface. However, following cotreatment with cPTH and trapidil, most of the mast cells were associated with osteoblasts. The receptor tyrosine kinase inhibitor gleevec prevented the cPTH-induced increase in peritrabecular mast cells (*B*). Wortmannin, a specific inhibitor of PI3K, dramatically decreased cPTH-induced mast cell localization at cancellous bone surfaces (*C*). Whereas trapidil had no effect on endocortical mast cells associated with osteoblasts in normal rats, a 1-week treatment with gleevec resulted in a dramatic decrease in the number of mast cells associated with osteoblasts (*D*). Values are mean ± SE. *Different from control, *p* < .05.

Compared with cPTH alone, cotreatment with cPTH and the PDGF receptor-α antagonist trapidil reduced the number of peritrabecular mast cells associated with fibroblasts by 90% (Fig. 5*A*). However, there was a 24-fold increase in the number of mast cells associated with osteoblasts. Thus trapidil did not lower the total number of mast cells recruited to bone surfaces. In contrast, the receptor tyrosine kinase inhibitor gleevec antagonized cPTH-induced mast cell relocalization to cancellous bone surfaces (Fig. 5*B*) and antagonized cPTH-induced osteitis fibrosa.(23) Wortmannin, a specific inhibitor of PI3K, also antagonized cPTH-induced mast cell relocation to bone surfaces (Fig. 5*C*), indicating that PI3K signaling is required for the mast cell response to cPTH.

The preceding studies focused on cPTH-induced increases in mast cell numbers at bone surfaces. We performed additional studies to determine whether trapidil or gleevec influences the number of mast cells already present on bone surfaces. In normal, nontreated rats, trapidil had no effect on endocortical distribution of mast cells, whereas a 1-week treatment with gleevec resulted in a dramatic decrease in the number of mast cells adjacent to endocortical osteoblasts (Fig. 5*D*).

### Studies in mice

**Fig. 6:**
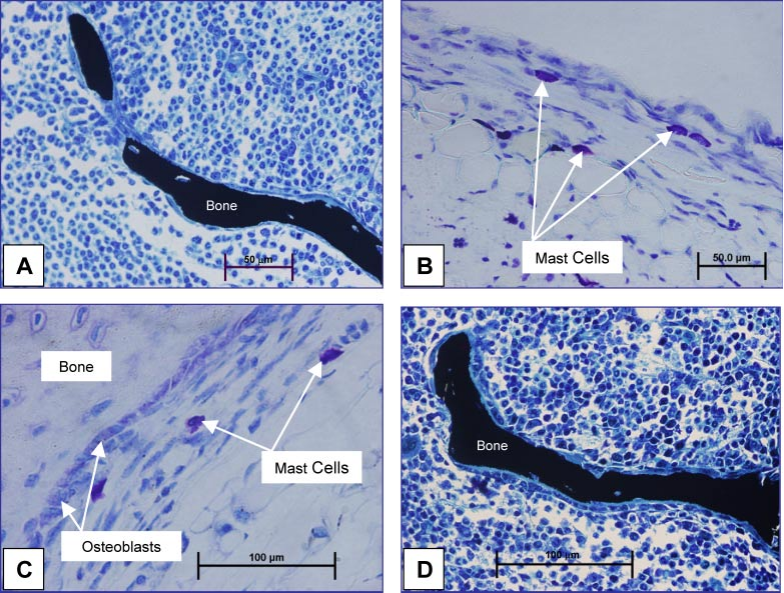
The distribution of mast cells in mice. Compared with rats and humans, mature mast cells in mice are very rare in bone marrow (*A*) but are common in skin (*B*) and periosteum (*C*). In contrast to rats, cPTH treatment in mice did not result in increased numbers of mast cells at endosteal bone surfaces and did not induce peritrabecular bone marrow fibrosis (*D*). Specimens shown in panels *A* and *D* are undemineralized, embedded in methyl methacrylate, and stained according to von Kossa with tetrachrome counterstain. Specimens shown in panels *B* and *C* are demineralized sections embedded in paraffin and stained with toluidine blue.

The effects of cPTH on mast cells and peritrabecular bone marrow fibrosis also were investigated in mice. Mature mast cells were not detected in bone marrow of control or cPTH-treated (1 and 2 weeks) DBA mice but were common in skin and detected at the periosteum (Fig. 6*A–C*). In turn, the DBA mice were resistant to the profibrotic effects of cPTH (Fig. 6*D*). These results (no cPTH-induced osteitis fibrosa) were confirmed in two other mouse strains (C57BL/6 and WBB6F1).

## Discussion

PTH, an important regulator of osteoblast and osteoclast differentiation, mobilizes hematopoietic stem cells in bone marrow and supports survival of hematopoietic stem cell progenitors.(30–32) Chronic HPT results in severe metabolic bone disease. In addition to the bone abnormalities described herein, HPT patients are anemic, have disturbed immune function,(33–36) and show a marked accumulation of hematopoietic-derived mast cells in their bone marrow.^37^ Mast cells play a key role in innate immunity. PTH increases mast cell number and secretion of mast cell–derived preformed effectors in rats.(38,39) To date, studies have emphasized the role of mast cell–derived histamine in osteoclastogenesis and bone resorption.(40–44) The role of mast cells in osteoblast differentiation and HPT-induced bone marrow fibrosis has received less attention.

**Fig. 7:**
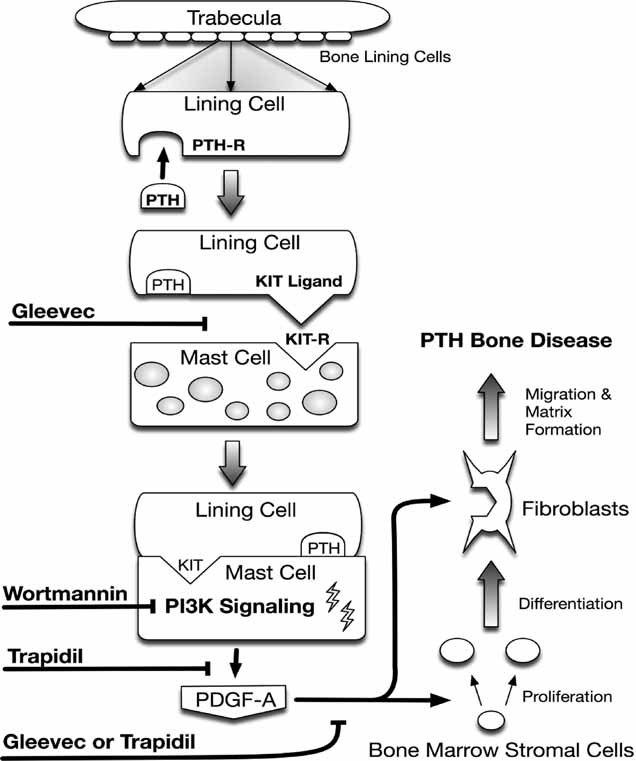
Model for the role of mast cells in the etiology of PTH-induced peritrabecular fibrosis. Chronic elevations in PTH levels result in the production of *kit* ligand by endosteal bone lining cells. *kit* ligand is chemotactic to mast cells, which aggregate at bone surfaces and overproduce PDGF-A. PDGF-A stimulates proliferation and differentiation of bone marrow stromal cells to preosteoblastic fibroblasts, which accumulate on endosteal bone surfaces and produce large quantities of poor-quality extracellular matrix that fails to mineralize properly. The initial aggregation of mast cells at bone surfaces can be blunted by the *kit* receptor antagonist gleevec as well as the PI3K antagonist wortmannin. PI3K is a major postreceptor mediator of *kit* signaling. The migration of preosteoblastic fibroblasts to bone surfaces in response to cPTH is also sensitive to trapidil, as well as gleevec, because both drugs are competitive inhibitors of the PDGF receptor-α. However, only gleevec prevents mast cell aggregation at bone surfaces.

Ellis and colleagues reported a 5-fold increase in bone marrow mast cells in iliac crest bone biopsies from patients diagnosed with HPT compared with healthy controls.(45,46) We confirmed this finding in HPT patients in this study. We also showed that elevated PTH increases the number of peritrabecular mast cells in rats. Blair and colleagues noted that mast cells in HPT patients were closely associated with osteoblasts.(37) Thus increased mast cell number is a consistent finding in humans with HPT and our rat model for HPT. As described in succeeding paragraphs, several independent lines of evidence support the conclusion that marrow mast cells may contribute to the excessive recruitment of osteoblast lineage fibroblasts to bone surfaces in parathyroid bone disease. A model for the actions of mast cells in response to PTH is summarized in Fig. 7.

Mast cells accumulate on endosteal (endocortical and cancellous) bone surfaces in renal failure patients, in rats following 5/6 nephrectomy, and in rats treated with cPTH. PTH receptor occupancy and mast cell accumulation at the bone–bone marrow interface appear to be essential, but not sufficient, for fibrosis. The PTH receptor ligand PTHrP, for example, increased bone resorption but was ineffective in increasing the number of mast cells on bone surfaces or inducing peritrabecular bone marrow fibrosis.(29) Osteitis fibrosa is rarely reported in patients with PTHrP-mediated hypercalcemia of malignancy, suggesting that mast cells play a role in mediating the differential skeletal responses to cPTH and PTHrP.

Gene profiling revealed that cPTH increases *PDGF*-A gene expression in femur,(47) and immunohistochemistry localized the PDGF-A peptide in cPTH-treated rats to mast cells.(16) Also, the PDGF receptor antagonist trapidil antagonized cPTH-induced peritrabecular fibrosis.(16) Thus increased peritrabecular mast cell numbers, increased *PDGF*-A gene expression, and PTH receptor occupancy appear to promote fibrosis.

Blair and colleagues interpreted the large increase in mast cells at the bone–bone marrow interface in patients with parathyroid bone disease as evidence for increased mast cell proliferation in response to osteoblast-derived stem cell factor (*kit* ligand).(37) However, our data, using an animal model, suggest an alternative possibility. We performed studies in which all cells progressing through the cell cycle were identified using a continuous [^3^H]thymidine labeling protocol.(14) These studies demonstrated that most of the fibroblasts recruited to endosteal surfaces in response to a 1-week treatment with cPTH had progressed through S phase of the cell cycle.(14) However, fewer than 1% of the mast cells were labeled with [^3^H]thymidine. Thus mature mast cells, or mast cell progenitors that could mature locally in the absence of further proliferation, appear to have been recruited to bone surfaces in response to cPTH.

The dramatic increase in *kit*-ligand expression observed by Blair and colleagues(37) provides a mechanism for accumulation of mast cells at the interface between bone and bone marrow in response to cPTH because *kit* ligand is a potent chemotactic factor for mast cells.(48) This hypothesis is supported by our observation that the *kit* antagonist gleevec antagonizes cPTH-induced mast cell redistribution and peritrabecular fibrosis.

Gleevec antagonizes signaling through c*-kit*, PDGF receptor-α, and the tyrosine kinase domain of the *Abelson* protooncogene (*abl*). Mice deficient in *abl* are osteoporotic and have defects in osteoblast maturation.(49) In contrast to c*-kit* and PDGF, there is no evidence that cPTH regulates *abl* signaling. The pharmacologic specificity of gleevec differs from that of trapidil, with inhibition of PDGF signaling being the only known overlap between the two drugs.

Gleevec promotes osteoblast differentiation by inhibiting PDGF receptor-α signaling.(50) On the other hand, trapidil inhibits cPTH-induced increases in skeletal PDGF receptor-α and *PDGF*-A gene expression.(47) Whereas both drugs inhibited cPTH-induced peritrabecular bone marrow fibrosis,(16,23) the present studies demonstrate that gleevec alone inhibits mast cell redistribution to bone surfaces. Taken together, these observations suggest that *kit* signaling facilitates peritrabecular localization of mast cells following treatment with cPTH, whereas peritrabecular localization of mast cells and PDGF-A promotes fibrosis.

Many of the downstream effects of *kit* ligand are mediated through PI3K signaling. In this study we showed that the PI3K inhibitor wortmannin prevents mast cell redistribution to bone surfaces in response to cPTH. We demonstrated previously that wortmannin reduces cPTH-induced peritrabecular fibrosis.(23) Together these findings suggest that PI3K signaling is required for cPTH-induced mast cell migration to bone surfaces. This conclusion is consistent with the well-established role for PI3K signaling in *kit*-dependent mast cell chemotaxis(48,51) but is contingent on the specificity of wortmannin for PI3K. Wortmannin has been shown to inhibit MAPK, but this action requires a concentration unlikely to be achieved in vivo.(52)

The effects of PTH have not been studied extensively in mice. Based on studies to date, it appears that mast cells are not required to elicit a skeletal response to the hormone. However, there are marked species differences in response to PTH. The dramatic fibrosis consistently observed in rats continuously infused with a comparable dose of PTH for 1 week was completely absent in the three mouse strains we have investigated. In mice, intermittent (once-daily) treatment with dose rates of PTH two orders of magnitude greater than used clinically elevates bone turnover and increases cortical bone mass by increasing periosteal expansion.(53,54) Despite increased bone turnover in mice treated with intermittent PTH, there was no change in cancellous bone volume.(53,55) In contrast, the major effect of a therapeutic (or higher) intermittent dose rate of PTH in rats(56) and humans is a net increase in cancellous bone mass.(57) Further studies will be necessary to establish whether the putative skeletal resistance of mice to PTH is due to the absence of mature mast cells on endosteal bone surfaces or other undefined factors.

The profibrotic effect of PDGF-A in soft tissues is well recognized.(58,59) For example, the growth factor has been implicated in the pathogenesis of liver fibrosis, a major determinant of the clinical course of chronic liver disease.(60) Also, idiopathic pulmonary fibrosis is characterized by alveolar macrophages that spontaneously release exaggerated amounts of PDGF-A, indicating that elevated PDGF-A plays a causative role in this condition.(20) Recent studies in our laboratory suggest that failure of preosteoblastic fibroblasts to differentiate into mature osteoblasts following their recruitment to bone surfaces plays a key role in the etiology of osteitis fibrosa.(14) On reaching bone surfaces, these preosteoblasts become positive for the osteoblast lineage transcription factor cbfa-1 and produce bone matrix proteins.(14) However, they maintain a fibroblast-like morphology, and the extracellular matrix they secrete fails to mineralize normally. The mineralization defect is due, at least in part, to overexpression of lysyl oxidase.(23) Further differentiation of the committed preosteoblastic fibroblasts appears to be blunted by the prevailing elevated PTH levels because parathyroidectomy in patients(61) and discontinuation of PTH infusion in rats(14) both result in a rapid disappearance of the fibrotic tissue and an increase in bone mass.

Immunolocalization studies suggest that cPTH results in overexpression of PDGF-A and lysyl oxidase by bone marrow mast cells. Coadministration of cPTH and the lysyl oxidase inhibitor BAPN or the PDGF-A signaling antagonist trapidil to rats blunted the mineralization defect and peritrabecular fibrosis, respectively.(23) Additionally, trapidil was shown to reduce osteoclast surface and hypercalcemia in rats treated with cPTH.(16) Thus there may be a mechanistic connection between peritrabecular fibrosis and focal bone resorption in patients with chronic HPT.

Although the present studies focus on pathological fibrosis, it is plausible that mast cells play a role in normal bone remodeling by regulating osteoblast recruitment and osteoclast function. This conclusion is supported by evidence that gleevec promotes osteoblast differentiation(50) and decreases mast cell number on bone surfaces.

In summary, mast cells are preferentially associated with bone surfaces at sites undergoing bone turnover, and their numbers on bone surfaces are regulated by PTH. Osteitis fibrosa, a hallmark of parathyroid bone disease, is associated with the increased presence of mast cells on bone surfaces, raising the possibility that the mast cell plays a role in the excessive recruitment of fibroblasts to bone surfaces. These findings indicate that the mast cell may represent a novel target for nonsurgical interventions to treat metabolic bone disease.
